# Strategies to Support Tobacco Cessation and Tobacco-Free Environments in Mental Health and Substance Abuse Facilities

**DOI:** 10.5888/pcd12.140585

**Published:** 2015-10-01

**Authors:** LaTisha L. Marshall, Nicole M. Kuiper, S. Rene Lavinghouze

**Affiliations:** Author Affiliations: Nicole M. Kuiper, S. Rene Lavinghouze, Office on Smoking and Health, Centers for Disease Control and Prevention, Atlanta, Georgia.

## Abstract

We identified and described strategies for promoting smoking cessation and smoke-free environments that were implemented in Oregon and Utah in treatment centers for mental illness and substance abuse. We reviewed final evaluation reports submitted by state tobacco control programs (TCPs) to the Centers for Disease Control and Prevention and transcripts from a call study evaluation. The TCPs described factors that assisted in implementing strategies: being ready for opportunity, having a sound infrastructure, and having a branded initiative. These strategies could be used by other programs serving high-need populations for whom evidence-based interventions are still being developed.

## Objective

Americans with mental illness, who often also have substance abuse problems, smoke at higher rates and die on average 25 years earlier than the rest of the US population (1,2). People with mental illness often lack access to treatment, and when they do gain access, they are often immersed in a culture that has the misperception that using tobacco is an appropriate way to cope with and manage their illness (3). Although national recommendations for smoking cessation treatment for this population exist, such as those of the American Psychiatric Association, these recommendations are not routinely implemented (3). Moreover, little is known about the progress of state tobacco control programs (TCPs) toward addressing this population. To disseminate information on innovative program models, we aimed to identify and describe the strategies used by TCPs in Oregon and Utah to support smoking cessation and tobacco-free environments in mental health and substance abuse facilities. 

## Methods

In 2010, TCPs in all 50 states, US territories, and Washington, DC, were awarded a total of $120 million (4) through the American Recovery and Reinvestment Act (ARRA) to decrease the prevalence of tobacco use (5). TCPs were permitted to choose the kind of intervention they wished to implement, and they were required to report their activities to the Centers for Disease Control and Prevention (CDC). In this study, we examined TCPs that reported on the use of these funds to integrate tobacco control efforts into facilities for treatment of mental illness and substance abuse.

We conducted a document review of final ARRA evaluation reports that were submitted by TCPs to CDC by September 1, 2013; 45 states submitted reports, and 4 states reported on a mental health component of their tobacco cessation intervention. Of these 4, we selected 2 states, Oregon and Utah, that included details on their programmatic work on smoking cessation and smoke-free environments in treatment facilities for mental illness and substance abuse during the 2-year ARRA funding period. We also reviewed transcripts from a 2011 call study evaluation (6). We identified and summarized the key innovative strategies used by the 2 programs.

## Results

Both TCPs, Utah’s Recovery Plus and Oregon’s Tobacco Freedom, used 3 key strategies: being ready for opportunity, having a sound infrastructure, and having a branded initiative.

**Being ready for opportunity.** TCPs in both states had been developing relationships with key partners for years before the ARRA funding; both had an action plan, and both were ready to implement activities that would further the plan’s progress among mental health and substance abuse populations. One TCP stated, “When the ARRA FOA [funding opportunity announcement] came out, it was the right fit, and we were ready to take advantage of the opportunity.” The other TCP had already been training quitline coaches to work with people with mental illness; the additional funding improved the ability of the quitline to identify and collect data on this population.

**Having a sound infrastructure.** Both TCPs discussed the importance of having sound infrastructure, which included support and buy-in from leadership at all levels and across agencies, champions (ie, strong supporters and promoters), data collection and use to assess tobacco-free policies and staff attitudes toward policies, and the ability to communicate and share success stories (Table).

**Table:** Examples of Strategies Used in Two States, Oregon and Utah, to Support Tobacco Cessation and Tobacco-Free Environments in Mental Health Facilities and Substance Abuse Facilities

Strategy	Example
**Multilevel leadership and champions**	
• Identify and develop long-term champions who are passionate about the tobacco-free initiative and can serve as a bridge to provider groups because they are known and trusted.	Leaders and champions are needed at all levels for collaborative initiatives to succeed and to ensure functioning program infrastructure and progress toward health goals (6,7). One program formed a leadership team that included clinical directors in substance abuse and mental health, representatives from local health departments, representatives from nonprofit organizations serving the mentally ill and substance addiction populations, champions, and clients. This team included both supporters and nonsupporters, because the program felt that “a team full of cheerleaders would not get us where we wanted to go.” The buy-in of the skeptics increased as they became informed about the excess illness and death among the mentally ill and substance abuse populations and realized that the program was focused on overall wellness. Having champions who can speak to misconceptions about clinical treatment issues was helpful in gaining credibility and support from clinical and medical directors.
• Institute a leadership team of multiple state agencies, which will promote cross-fertilization of ideas and provide input on how to operationalize the strategies needed.	
• Have a tobacco-control program (TCP) staff position responsible for working closely with the substance abuse and mental health agency to keep all partners connected and to facilitate communication.	
**Collection and use of data**	
• Assess needs and support for smoke-free policies of facilities and providers through needs assessments or surveys to determine their current policies or attitudes toward agency policies.	Data can be used in a manner that engages partners to act (6,7). The assessments were done through client focus groups and key informant interviews with staff from various publicly funded substance abuse and mental health treatment centers. These data informed TCPs of centers’ tobacco control policies, client and staff attitudes about tobacco use, readiness to change policies, and barriers they would face in becoming smoke-free; the data also provided information on current training needs of staff. These data allowed TCPs to understand the scope of the issue in their state and target their interventions appropriately. As a result, embedding tobacco use questions within the patient intake system enabled substance and mental health treatment centers to identify clients who were smokers. This identification gave TCPs the ability to track smoking, the opportunity to refer patients to the state quitline, and the opportunity to offer cessation treatments or to identify those already receiving treatment.
• Enhance quitline data collection to properly identify and collect data on mental health populations.	
**Planning**	
• Network with other successful programs to plan your program.	Plans should be dynamic and evolve in response to the leadership team, context, priorities, and scientific evidence (6,7). For both state programs, a plan to work in mental health and substance abuse treatment centers, as well as relationships with potential partners, had already been developed. Therefore, the programs were able to act swiftly when the funding opportunity became available.
• Integrate with other chronic disease program areas to incorporate other health-related activities as part of the recovery process, such as measuring weight and height upon intake, calculating body mass index, offering nutrition classes, and creating more opportunities for physical activity.	
• As a part of protocol planning, have a coordinator at the mental health facility who will take responsibility for registering with the quitline, coordinating counseling calls, and receiving and dispensing nicotine replacement medications.	
**Training**	
• Train mental health facility owners, managers, and staff and local health department staff on misperceptions about tobacco use among people with mental illness, tobacco cessation, and policy implementation to build the capacity of the internal staff and partners in providing a shared understanding of tobacco use among people with mental illness.	Training, technical assistance, and follow-through are necessary to ensure the proper use of data and implementation of policies (6,7). One program used the data collected during assessments of substance abuse and mental health facilities to provide outreach and technical assistance; peer-to-peer counseling and resources were included.
**Communication**	
• Organize a media program that promotes success stories about real clients who quit smoking while in recovery in a tobacco-free facility.	One program had a news media event in which leadership from both the TCP and substance abuse and mental health showed their commitment to supporting tobacco cessation as a part of treatment. In addition, a website was created to focus on tobacco-cessation activities among the mental health and substance abuse population.

**Having a branded initiative.** Both TCPs had branded initiatives that gave their project a recognizable name. Recovery Plus aimed at promoting health and wellness among people with mental illness or substance abuse (8); Tobacco Freedom aimed to improve access and participation in tobacco cessation treatment plans as part of mental health and addiction services (9). One TCP met 3 objectives of their initiative: 1) creating 100% tobacco-free campuses for addiction or mental health treatment centers and clinics, 2) having a policy that requires a cessation plan upon client discharge, and 3) having a policy that prohibits staff members from providing tobacco to clients. The other TCP enacted a 100% tobacco-free campus policy for publicly run treatment facilities.

## Discussion

Smoking is entrenched in the culture of mental health and substance abuse; it will take time to change this culture. TCPs in Oregon and Utah engaged partners treating substance abuse and mental illness in the process of change and built trust along the way. These partnerships created an opportunity for partners to participate in developing a realistic and feasible plan for applying the evidence base for tobacco cessation and treatment to a population that has a disproportionate share of smoking-related illness and death.

The 2 TCPs were positioned to increase access to tobacco cessation services among people with mental illness because they were ready for opportunity, had a sound infrastructure, and had a branded initiative. Buy-in and ownership of the initiative from leadership and partners were seen not only as critical for implementation success but also for sustainability (6,7). This level of engagement was created by identifying champions, sharing client success stories, and ensuring that stakeholders, including community members, clients, and other partners, were involved throughout the engagement process.

Collecting and using data allowed TCPs to assess policies and agency attitudes toward policies (10) and to evaluate the training needs of staff members serving this population. Adding tobacco use questions to the client intake system gave mental health centers the ability to identify clients who were smokers. This identification allowed the TCPs to track tobacco use, refer clients to the state quitline, and offer cessation treatments.

Utah and Oregon received funding to continue their initiatives. Other state TCPs interested in addressing tobacco use among the mentally ill might consider integrating the strategies described in this article into their systems to improve tobacco cessation efforts and promote smoke-free environments in facilities serving this population. Although the effectiveness of these strategies has not been assessed, Utah and Oregon provide program models that could be used among high-need populations in which best practices are not yet documented or evaluated. Moreover, the experience and lessons learned in Utah and Oregon provide an opportunity to share what has been done and the potential to reduce tobacco-related morbidity and mortality among those with mental illness or substance abuse disorders.
